# The Effect of Oxidative Modification of Activated Carbon on Adsorption of Aromatic Compounds from Aqueous Solutions

**DOI:** 10.3390/molecules30183810

**Published:** 2025-09-19

**Authors:** Anna Derylo-Marczewska, Andrzej Swiatkowski, Grzegorz Trykowski, Stanislaw Biniak

**Affiliations:** 1 Institute of Chemical Sciences, Faculty of Chemistry, Maria Curie-Sklodowska University, M. Curie-Sklodowska Sq. 3, 20-031 Lublin, Poland; 2 Institute of Chemistry, Military University of Technology, Kaliskiego 2, 00-908 Warsaw, Poland; a.swiatkowski@wp.pl; 3 Faculty of Chemistry, N. Copernicus University, 87-100 Torun, Poland; tryki@chem.umk.pl (G.T.); sbiniak@chem.umk.pl (S.B.)

**Keywords:** activated carbon, surface chemistry, adsorption of organics

## Abstract

Activated carbon F-400 was modified by using different oxidation agents: nitric acid, sulfuric acid, and ozone. The influence of the type of carbon surface groups on adsorption effectiveness towards selected aromatic compounds was analyzed. The commercial carbon F-400 was deashed and modified, and the obtained materials were characterized by using different techniques to determine their textural, thermal, morphological, and surface properties: low-temperature adsorption/desorption isotherms of nitrogen, scanning electron microscopy (SEM), Boehm titration, and Fourier transform infrared spectroscopy (FTIR). The adsorption properties towards four aromatic compounds, i.e., toluene, 4-nitrotoluene, nitrobenzene, and 4-nitrobenzoic acid, were evaluated based on isotherm measurements. Adsorption equilibrium data were analyzed by applying the generalized Langmuir isotherm. The influence of carbon surface groups and adsorbate functional groups in interaction mechanisms was discussed. It was found there was a strong effect of oxidation on adsorption efficiency. The adsorption capacity of modified activated carbon F-400 strongly depends on the type of oxidant used, e.g., in the case of toluene, the use of ozone as an oxidant gives a sorption capacity of 5.51 mmol/g, and of nitric acid—4.20 mmol/g.

## 1. Introduction

Carbons with well-developed porosity are successfully used in many applications, such as adsorption, separation, environmental remediation, catalysis, drug delivery, and energy storage. The effectiveness of activated carbons in the processes of water and wastewater treatment depends on the properties of the adsorbent, adsorbate, and solution, as well as the conditions of establishing adsorption equilibrium. The structural and surface characteristics of the adsorbent affect the adsorption process to a large extent; the effect of surface properties especially is very important in adsorption from liquids [1,2,3,4,5,6,7,8,9]. The surface characteristics of carbons depend on the properties of their surface functional groups determining the charge, hydrophilic or hydrophobic behavior [7], and electronic density of the graphene layers [5]. Surface oxygen functional groups play a particularly important role. Many of their types are known, as are numerous methods for their formation on the surface of carbon materials. The use of appropriate methods, oxidizing substances, and process conditions makes it possible to regulate the amount and type of oxides formed on the surface [9,10,11,12,13,14,15,16,17,18,19,20,21,22,23,24,25,26,27,28,29,30].

Surface oxygen functional groups play a particularly important role. In the case of granulated activated carbons, a problem with unequal oxidation levels within the granule can also be observed. The further away the pore walls are from the outer surface of the granule and closer to its center, the less oxygen is chemically bound during the oxidation process [10]. Another way to regulate the amount and type of oxygen functional groups present on the surface of the carbon material is to strongly oxidize it and then to control their partial thermal decomposition by heating the carbon material in a vacuum or inert gas atmosphere [8].

Many oxidizing substances are known to be used in the process of generating oxygen surface functional groups on the surface of various carbon materials, e.g., activated carbons [9]. The nature of the adsorbate substituent also affects the interactions in the adsorption system, resulting in decreasing or increasing adsorption value [2]. Generally, oxidation processes of activated carbons are important due to their applications in multicomponent systems containing oxidizing substances. The presence of such compounds can significantly reduce the adsorption efficiency of hydrophobic organic compounds. Conversely, oxidation, which increases the hydrophilicity of the surface, can also increase the adsorption of hydrophilic compounds.

The aim of this paper is to estimate the dependence between the surface characteristics of unmodified and modified activated carbons and the character of the adsorbate functional group on adsorption from dilute aqueous solutions. The commercial activated carbon F-400 was deashed and modified to differentiate its surface properties. These involved subsequent oxidation with various oxidants (in the liquid and gas phases). The obtained samples were studied to characterize their physicochemical and adsorption properties. The adsorption effectiveness of the modified carbons towards selected aromatic compounds containing a benzene ring but different functional groups (-CH_3_, -NO_2_, -COOH) was studied based on equilibrium data. The experimental isotherms were analyzed to find the correlations between adsorption efficiency and surface properties. Such a large group of adsorbent–adsorbate systems (20 systems) allowed us to assess the influence of various factors on the adsorption process.

## 2. Results and Discussions

### 2.1. Carbon Characterization

**Figure 1:**
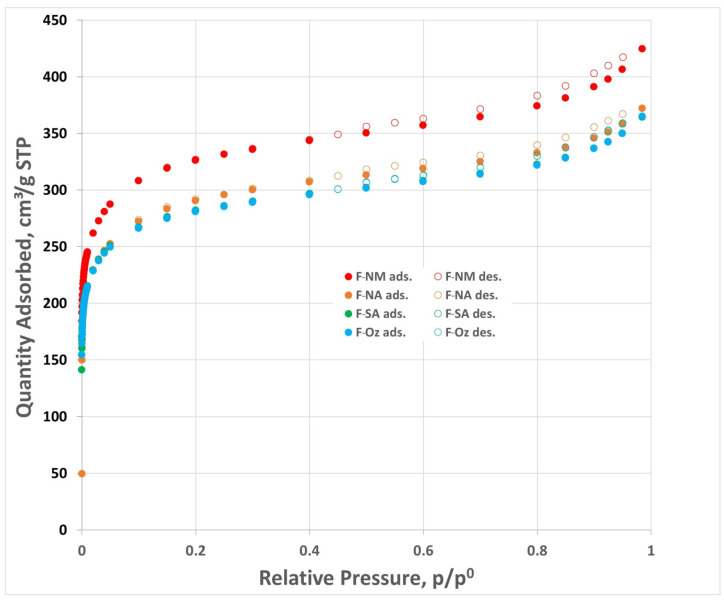
Low-temperature nitrogen adsorption–desorption isotherms on carbon F-NM (deashed commercial F-400 granulated activated carbon) and modified materials F-NA (oxidized with nitric acid), F-SA (oxidized with sulfuric acid), and F-Oz (oxidized with ozone). (ads—data from adsorption branch, des—data from desorption branch).

The textural properties of the deashed and oxidized carbons were determined based on the low-temperature nitrogen adsorption–desorption isotherms, which are shown in Figure 1. All isotherms represent type I according to IUPAC classification, typical for microporous materials. The F-NM (deashed F-400) activated carbon is characterized by the most highly developed porosity. The remaining samples modified in oxidation processes show slightly weaker adsorption properties, and the shapes of nitrogen isotherms are very similar. The values of parameters characterizing textural properties of the studied materials, determined from nitrogen isotherms, are summarized in Table 1. It is evident that the porous structure (represented in Table 1 by the BET specific surface area, S_BET_, micropore volume, V_mi_, total pore volume, V_t_) changes rather slightly in oxidation processes.

Applied modification methods differentiate the surface chemistry of activated carbons. In Table 1, the values of selected parameters characterizing the surface and thermal properties of the modified carbon samples are summarized. One can find some correlations between the parameters characterizing chemical properties of the carbon surface; for example, the lower is pH of carbon slurry, the higher the uptake of NaOH and a relative mass loss in the temperature range 150–400 °C (thermal decomposition of carboxylic groups) or 400–600 °C (decomposition of lactone groups) [25]. All three modification methods result in an increase in surface acidity (amount of surface oxygen groups). The most oxidized carbon is the F-NA sample. Both remaining samples (F-SA and F-Oz) show much lower oxidation levels.

**Figure 2:**
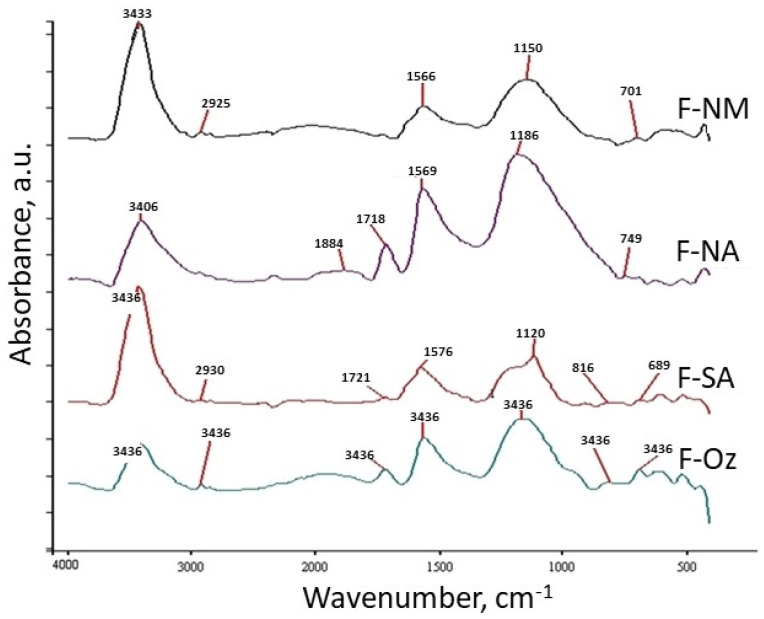
Absorption (a.u.—arbitrary units) FTIR spectra of deashed carbon F-NM and modified materials F-NA, F-SA, and F-Oz.

The FTIR spectra illustrated in Figure 2 give more precise information on surface chemistry. All the FTIR spectra (Figure 2) exhibit a band with a maximum at 3430 cm^−1^, characteristic of the -OH moiety (structural hydroxyl functional groups and adsorbed water molecules). The asymmetry of these peaks (i.e., the tail and the convolutions on the lower wave number side) may indicate strong hydrogen interaction with adsorbed water molecules. The bands at about 2930 cm^−1^ are related to the CH groups. Below 2000 cm^−1^, the FTIR spectra of non-modified carbon (F-NM) show absorption typical of surface and structural oxygen species. The presented spectra are similar to the reported spectra of other activated carbons derived from a variety of organic precursors by carbonization and activation in the gas phase. The presence of overlapped bands at the 1630–1560 cm^−1^ range can be attributed to stretching vibrations of C=O moieties in surface quinone (ketone), enol, cyclic b-ketones, and/or iono-radical structures, as well as conjugated systems like diketone, keto-esters, and keto-enol structures. The complicated nature of the adsorption bands in the 1650–1500 cm^−1^ region suggests that aromatic ring bands and double bond (C=C) vibrations overlap the aforesaid C=O stretching vibration bands and OH binding vibration bands. The partially resolved peaks forming the absorption band in the 1260–1000 cm^−1^ region can be assigned to ether-like (symmetrical stretching vibrations), epoxide and phenolic vibrations (peak maximum at 1150 cm^−1^), and similar structures existing in different structural environments. These observations are confirmed by mass loss of F-NA and F-Oz samples mainly in the temperature range 150–400 °C and partially 400–600 °C (Table 1), and according to [25].

The FTIR spectra of the oxidized carbons (F-NA, F-Oz) are quite similar to those obtained for various carbon materials oxidized with nitric acid or dioxygen. After oxidation, the intensity of the band characteristic of carbonyl moieties in a carboxylic acid (near 1720 cm^−1^) increases, and there is a simultaneous, considerable increase in band relative intensity of these carbonyl functional groups (in the wavenumber range between 1600 and 1500 cm^−1^) in different surroundings [25]. Additionally, a new band characteristic of carbonyl moieties in carboxylic anhydrides appears in the 1850–1800 cm^−1^ region. Oxidation also changes the shape of the overlapping bands in the “fingerprint” region (1400–1000 cm^−1^), mainly enhancing relative absorption in the 1250–1000 cm^−1^ range (maximum near 1180 cm^−1^) [25]. This suggests an increase in the number of ether and hydroxylic structures on the carbon investigated. It is confirmed by mass loss of F-NA and F-Oz in the temperature range 400–600 °C (Table 1) and according to [25].

As a result of the modification of the carbon samples by concentrated sulfuric acid treatment (F-SA), the relative increase in C=O and C-O-C bands is smaller than for the after-oxidation modification. One additional band (with a maximum at 1120 cm^−1^) with several overlapping bands present in the 1400–1050 cm^−1^ range can be attributed to the surface sulphonic acid groups (>C-SO_2_OH).

**Figure 3:**
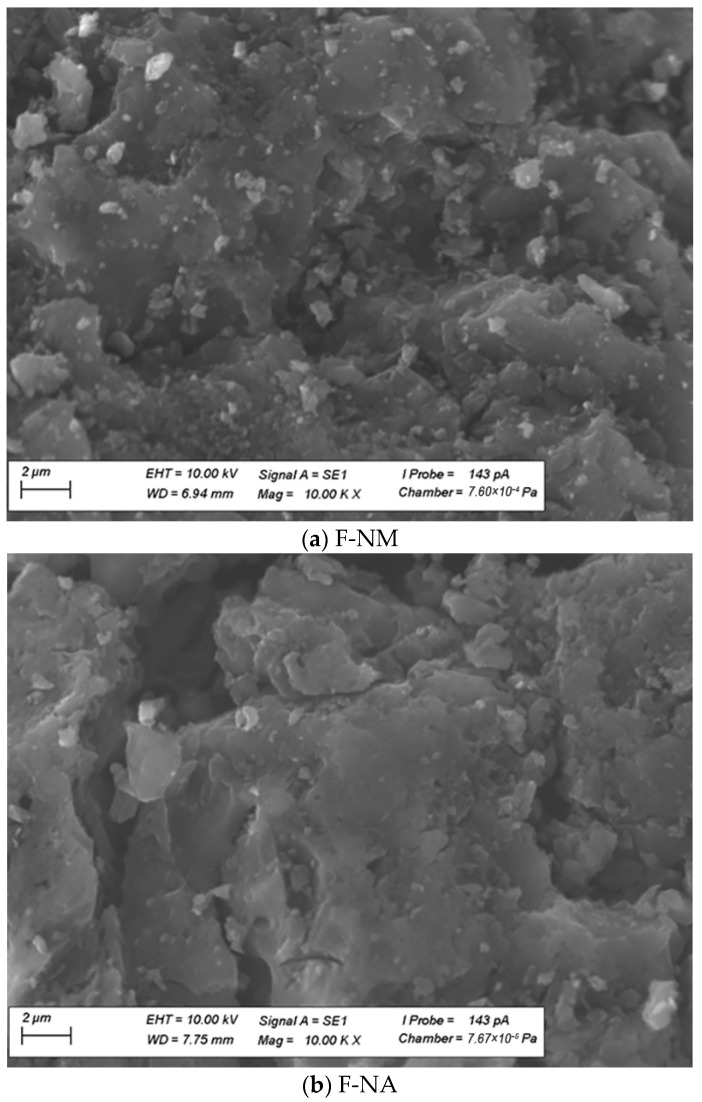

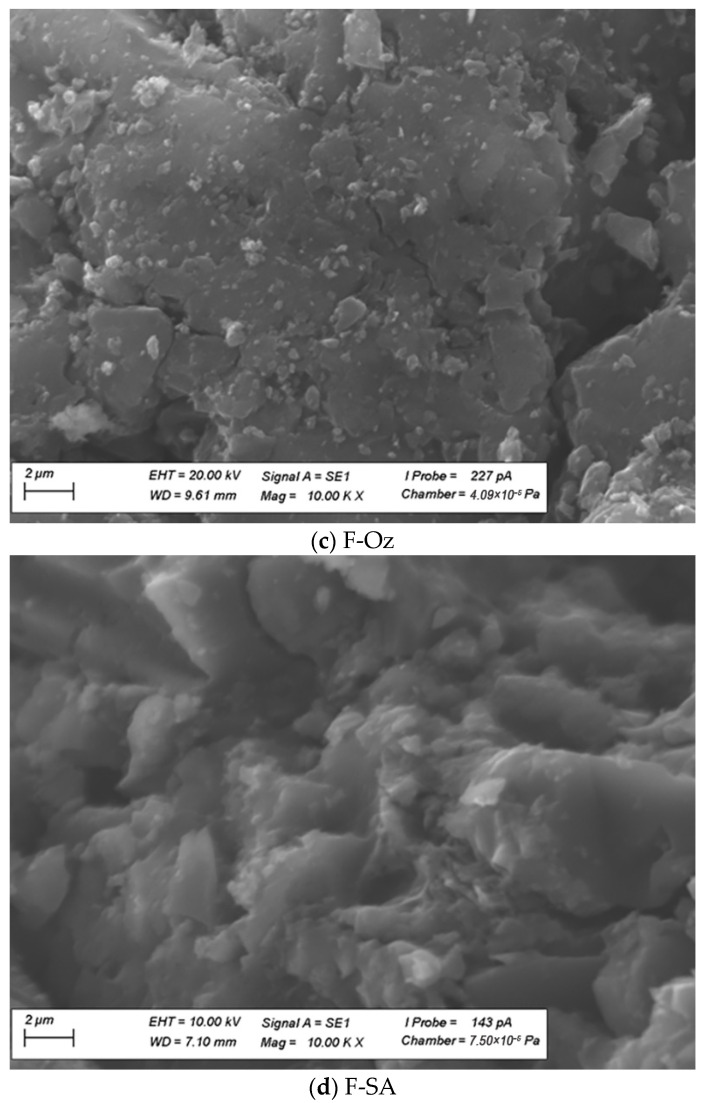
SEM images (magnification 10.00 KX) of modified activated carbon F-400 samples: (**a**)—F-NM, (**b**)—F-NA, (**c**)—F-Oz, and (**d**)—F-SA.

Additionally, to illustrate the differences in the surface morphology of the F-400 modified activated carbon samples, SEM images were selected and presented in Figure 3. Comparison of the effects of the oxidation methods used shows that when concentrated nitric acid was used, many of the fine particles (2–5 nm) present on the surface of the F-NM sample were dissolved. A similar effect was observed when concentrated sulfuric acid was used as the oxidizing agent. This effect was not observed when ozone was used in the oxidation process.

To better characterize the surface chemistry of modified F-400 activated carbon preparations, the chemical composition of their surfaces was determined using SEM-EDS. The obtained results are presented in Table 2. The data presented in Table 2 indicate particularly large differences in oxygen content.

### 2.2. Adsorption Properties of Modified Carbons

**Figure 4:**
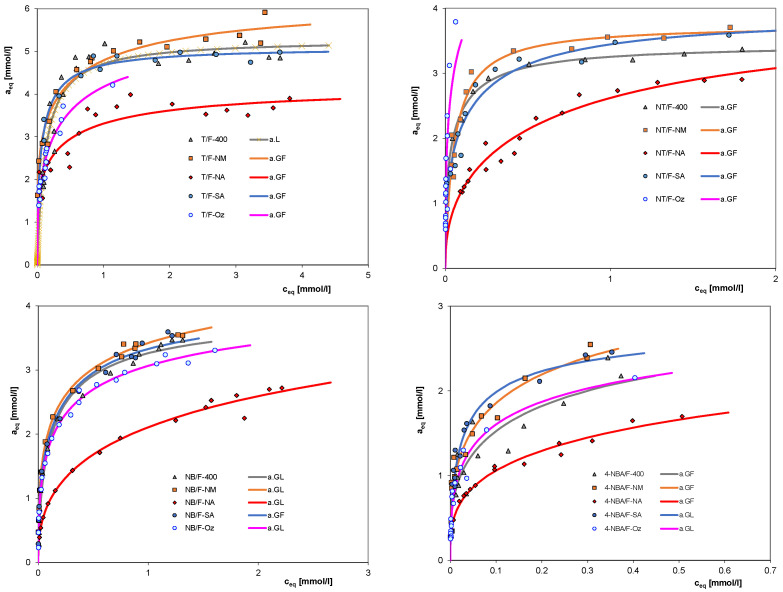
Comparison of adsorption isotherms of toluene (T), 4-nitrotoluene (NT), nitrobenzene (NB), and 4-nitrobenzoic acid (4-NBA) from aqueous solutions on various carbons: F-400, F-NM, F-NA, F-SA, and F-Oz (influence of adsorbent properties).

The chemical structure of activated carbon surfaces and the properties of the adsorbate are crucial in establishing adsorption equilibrium at the solid/liquid interface. These factors often outweigh the textural properties of the adsorbents. To study the influence of carbon surface chemistry on adsorption effectiveness, the measurements of isotherms were performed for aqueous solutions of toluene (T), 4-nitrotoluene (NT), nitrobenzene (NB), and 4-nitrobenzoic acid (4-NBA) and the following carbons: commercial activated carbon F-400, deashed carbon F-NM, and modified carbons F-NA, F-SA, and F-Oz. The selected physicochemical properties of used adsorbates are presented in Table 3. The experimental measurements allowed us to analyze the influence of both the presence of carbon surface groups with different properties and the structure of the adsorbate molecule (the size and nature of the functional group on the aromatic ring) on the efficiency of adsorption from the aqueous phase. Figure 4 presents the adsorption isotherms of four selected aromatic compounds on all the activated carbons tested.

Let us start with the analysis of adsorption isotherms of toluene on all studied activated carbons (Figure 4). Toluene is a highly volatile compound, and therefore, the experimental results are subject to significant measurement error. However, we can easily find that toluene adsorbs least strongly on the F-NA carbon, while the adsorption isotherms for the three carbons—F-400, F-NM, and F-SA—show similar behavior. The F-Oz carbon is characterized by medium adsorption efficiency, except for the system with nitrotoluene as the adsorbate. For the remaining adsorbates, the weakest adsorption was also observed on the F-Na carbon, oxidized with nitric acid. However, in almost all cases, the highest adsorption affinity is shown by the deashed F-NM activated carbon, and slightly lower by the commercial F-400 carbon, which is related to the larger specific surface area and pore volume of the F-NM material. The relatively highest adsorption observed for the F-NM sample is connected with the deashing process, which can make some of the previously blocked micropores accessible, thus increasing the overall pore volume and adsorption capacity of this material. The starting carbon, containing some ash, and the F-SA carbon, modified with sulfuric acid and thus containing some surface functional groups, exhibit slightly reduced sorption properties. The above conclusions show a general trend of decreasing adsorption efficiency in the case of carbons oxidized with nitric acid; however, when using other oxidants, the adsorption efficiency shows variable behavior, which should be related to both the chemical nature of the surface groups and the properties of the adsorbates.

The oxidation process leads to a reduction in the adsorption of organic substances; the type of modification used significantly affects the adsorption efficiency. The weakest adsorption of all tested aromatic compounds on the F-NA carbon is due to the presence of a large number of oxygen groups, formed as a result of oxidation of the adsorbent with concentrated nitric acid. The F-NA carbon, on which the selected aromatic compounds adsorbed the least, contains the most acidic functional groups. The adsorption of aromatic compounds on activated carbons is dominated by dispersive interactions between the π electrons of the aromatic ring of the adsorbate molecule and the graphene planes of the activated carbon. Surface oxygen compounds influence the dispersive interactions, determining the mechanism of the adsorption process. Surface oxygen groups cause changes in electron density in the graphene planes by attracting and localizing π electrons from these planes. The resulting “+” holes reduce the interactions between the carbon and the aromatic ring of the adsorbate. Aromatic compounds adsorb well on carbons with basic groups due to the existence of electron-rich Lewis base centers. The possible mechanism of adsorption reduction is also the accumulation of water clusters on the functional (oxygen) groups, blocking access to the micropores and some of the active sites. In the case of carbons rich in surface functional groups, other interactions stronger than dispersion should also be taken into account, such as the charge transfer mechanism or the electron donor–acceptor mechanism.

Therefore, in systems composed of oxidized activated carbon and aqueous solutions of organic substances, the following interactions should be taken into account: reduction in the hydrophobicity of the carbon surface, changes in electron density in the carbon planes, formation of hydrogen bonds between surface oxygen groups and water molecules, and resulting changes of the adsorption mechanism. These effects lead to reduced adsorption on the carbon oxidized by nitric acid. However, such effects are not observed for the materials obtained by modification with sulfuric acid and oxygen/ozone mixture treatment. Taking into account oxygen content, one can find that the F-NA carbon is the richest with O_2_ (17.54 wt.%), while the F-Oz sample contains a slightly lower amount (17.34 wt.%); thus, they should show similar adsorption effectiveness towards the same solutes. However, as shown by the results of Boehm titration and FTIR spectra, the nature of the surface groups introduced during oxidation with nitric acid and ozone is diverse; hence, different adsorption mechanisms determine the different adsorption efficiency of both carbons.

Another effect examined was the effect of aromatic adsorbate properties on the adsorption efficiency from the aqueous phase onto activated carbon. When analyzing the effect of an adsorbate on the establishment of adsorption equilibrium in this system, one must consider several factors: its hydrophobicity (solubility), molecular size, the number and properties of functional groups on the aromatic ring that interact with the carbon surface differently, the possible effect of steric exclusion of larger molecules from the smallest pores, and the molecular state of compounds capable of dissociation (Figure S1, Supplementary Materials). Generally, analyzing the isotherms in terms of the influence of adsorbate properties, one can state that in the case of four carbons—F-400, F-NM, F-NA, and F-SA—similar effects were observed. Analyzing the plotted isotherms, we note significantly higher adsorption of toluene compared to the adsorption of other compounds. For these systems, the adsorption effectiveness decreased as follows: T > NT > NB > 4-NBA, while for the carbon F-Oz, the highest adsorption was found for nitrotoluene. In the case of adsorption of organic compounds from aqueous solutions, the driving force behind the adsorption process is the hydrophobicity of the adsorbate; the lower the solubility of a substance, the stronger its adsorption on the hydrophobic surface of activated carbon. Comparing the solubility values of the tested compounds listed in Table 3, one should conclude that 4-nitrobenzoic acid has the highest affinity for carbon materials due to its lowest solubility. Two other adsorbates, 4-nitrotoluene and toluene, are characterized by slightly higher solubility. However, when analyzing the adsorption isotherms of these compounds from the aqueous phase on microporous carbons, in addition to the hydrophobic–hydrophilic properties, the role of the functional group and the molecular size of the adsorbate should also be considered due to the sieve effect. The overlap of these effects results in the course of the experimentally observed adsorption isotherms.

**Figure 5:**
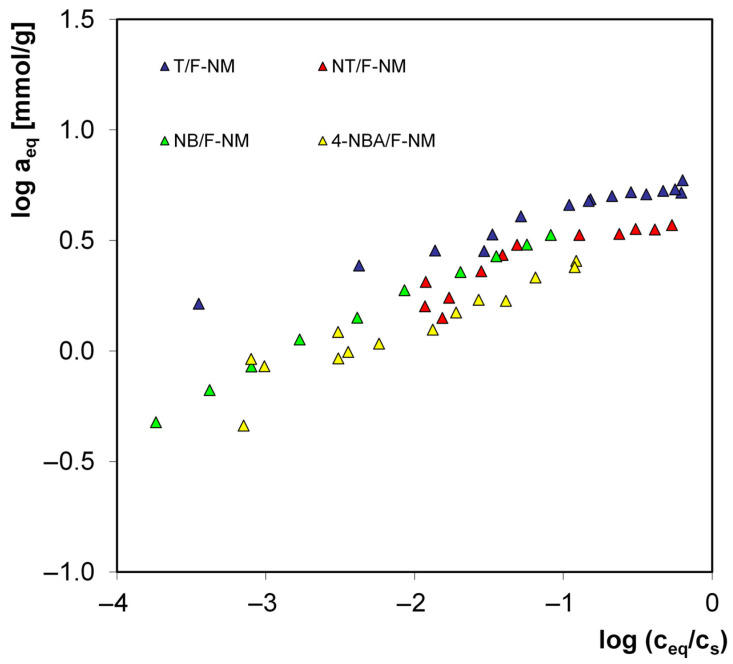
Comparison of adsorption isotherms of toluene (T), 4-nitrotoluene (NT), nitrobenzene (NB), and 4-nitrobenzoic acid (4-NBA) from aqueous solutions on F-NM carbon.

To assess the influence of the functional group properties of aromatic compounds on the efficiency of the adsorption process, the experimental isotherms may be analyzed in the reduced system (adsorption as a function of equilibrium concentration divided by solubility, c/c_s_), thus eliminating the influence of differences in their hydrophobicities. Presenting adsorption isotherms in this reduced system allows for the examination of the actual affinity of a given type of compound for the carbon surface due to the properties resulting from the specific functional group. Figure 5 shows example isotherms in the reduced coordinate system for the adsorption of various adsorbates on the best F-NM carbon (similar results were obtained for the other adsorbents). The strongest affinity is observed for toluene and the weakest for 4-nitrobenzoic acid. Toluene possesses a –CH_3_ functional group that weakly activates the aromatic ring in the ortho and para positions, which shifts electrons toward the ring, thus enhancing the interactions of the toluene molecule with the π electrons of the graphene carbon planes. On the other hand, 4-nitrobenzoic acid has two functional groups, COOH and NO_2_, which strongly deactivate the aromatic ring, reducing the electron density in the ring and thus weakening interactions with the carbon surface. In the case of nitrobenzene, the deactivating nitro group occurs. The relatively high affinity, greater than for 4-nitrotoluene (two groups with opposite, canceling out properties), is probably due to steric considerations.

For analysis of experimental adsorption data, the generalized Langmuir equation (GL) (Equation (2)) was applied. By using the non-linear fitting procedure, the values of parameters were obtained and presented in Table 4. The determination coefficients (R^2^:0.81–0.99) evidence measurement errors in the case of some adsorbates showing low solubility or high volatility. Furthermore, it is worth commenting on certain discrepancies that may arise when comparing the adsorption isotherms and the adsorption capacity values obtained using the optimization method. This is due to larger experimental errors for poorly soluble and highly volatile substances and the relatively narrow range of equilibrium concentrations, which means that the adsorption capacity obtained from the GL equation may be underestimated or overestimated due to the mathematical characteristics of the isotherms.

Based on literature data, Table 5 compares the effect of carbon oxidation on the adsorption efficiency of selected aromatic compounds for various systems. Comparing the adsorption capacities of selected adsorbates, several regularities can be observed. Ash removal always increases adsorption capacity. The use of nitric acid as an oxidant for deashed activated carbon most often causes a significant reduction in the adsorption capacity of similar adsorbate types (phenol, o-chlorophenol, p-nitrophenol, aniline [29], and phenol, aniline, nitrobenzene [30]). In general, a reduction in adsorption capacity can be observed for carbon materials oxidized using various oxidants.

## 3. Materials and Methods

### 3.1. Chemicals and Materials

Activated carbon Filtrasorb F-400 was purchased from Chemviron (Feluy, Belgium). Toluene, 4-nitrotoluene, nitrobenzene, and 4-nitrobenzoic acid were bought from Avantor Performance Materials Poland S.A. (Gliwice, Poland). 

### 3.2. Carbon Sample Preparation

Samples of demineralized with concentrated HF and HCl acids (0.1% ash) and next modified F-400 (Chemviron) activated carbon were used as adsorbents. The demineralized carbon (F-NM) was modified in different ways: oxidation with concentrated (65%) nitric acid, treatment with concentrated (98%) sulfuric acid, and oxygen/ozone mixture treatment.

F-NA carbon sample—F-NM oxidized with concentrated (65%) HNO_3_ at 356 K, time 3 h, after which the sample was washed with demineralized H_2_O until pH ~7 and the NO_3_^−^ ions were completely washed out.

F-SA carbon sample—F-NM oxidized with 98% H_2_SO_4_ at a temperature of 373 K, time 3 h, after which the sample was washed with demineralized H_2_O until pH ~7 and SO_4_^2−^ ions were completely washed out.

In the case of the F-Oz carbon sample, the method consisted of passing a stream of ozone through a fluidized bed of carbon F-NM at a temperature of 293 K for 3 h. The ozone was produced in a generator with a capacity of 15 g O_3_/h.

After the modification procedures, all the activated carbon samples were dried to constant mass at 373 K.

### 3.3. Carbon Sample Characterization

The porous structure and surface characteristics of the carbons were estimated by applying various experimental methods: the low-temperature nitrogen adsorption/desorption isotherms, the Boehm method, the thermogravimetric and FTIR analysis, and the elemental analysis.

Nitrogen adsorption/desorption isotherms at 77.4 K were determined by using an ASAP 2010 analyzer (Micromeritics, Norcross, GA, USA). From the nitrogen adsorption data, the values of the BET specific surface area of the carbon samples were calculated.

The chemical properties of the modified carbon surface were determined by the standard neutralization—titration with HCl or NaOH (0.1 M aqueous solutions) according to the Boehm procedure. Additionally, the pHs of carbon slurries in 0.1 M NaCl were determined.

To investigate the differences in chemical properties of the studied carbons, the thermogravimetric (TG) measurements were also performed using the thermoanalyzer Universal V3.0 G TA Instruments (New Castle, DE, USA). The carbon samples were heated from 25 °C to 1000 °C (10 °C/min) in an argon atmosphere. From the obtained TG curves, the relative mass loss (Δm/m) values in temperature ranges 150–400 °C and 400–600 °C were calculated.

The type and concentration of oxygen-containing groups on the surface of activated carbon were recorded spectroscopically (FTIR). Carbon–KBr mixtures (1.5:300) were ground, then desorbed/degassed at room temperature to obtain IR-transparent pellets. The absorbance FTIR spectra (FTIR Spectrum 2000 spectrometer—Perkin-Elmer, Waltham, MA, USA) were recorded in the range 4000 to 400 cm^−1^ at a scan rate of 0.2 cm s^−1^, and the number of interferograms at a nominal resolution of 4 cm^−1^ was fixed at 25. Before the spectrum of a sample was recorded, the background line was obtained arbitrarily and subtracted. The IR measurements applied here (KBr-pellet technique) make it impossible to compare quantitatively the FTIR spectra obtained for different carbons, but they can indicate which individual chemical structures may or may not be present in the carbon.

The morphological analysis of the materials obtained was possible through advanced electron microscopy techniques. These studies used the scanning electron microscope (SEM, LEO 1430 VP, Leo Electron Microscopy Ltd., Cambridge, UK) coupled with energy-dispersive X-ray detection (EDX) (XFlash 4010, Bruker AXS, Berlin, Germany).

### 3.4. Adsorption Isotherm Measurements

Adsorption isotherms were measured for all studied carbons: F-400, F-NM, F-NA, F-SA, F-Oz, and four organic adsorbates: toluene (T), 4-nitrotoluene (NT), nitrobenzene (NB), and 4-nitrobenzoic acid (4-NBA). All measurements were performed at a temperature of 293 K, in a solution with an ionic strength of I = 0.1 and a constant pH of 2.2. Under these conditions, the molecules of dissociable adsorbates were present in the solution in a non-ionized form. Adsorption processes were carried out in Erlenmeyer flasks into which a specific amount of activated carbon and a constant volume of adsorbate (*V* = 100 mL) of specific concentration were introduced. The detailed values were as follows: for toluene—*c_o_* = 2.8–4.705 mmol/L, *m* = 0.02–0.1 g; for nitrotoluene—*c_o_* = 1.5564–2.4877 mmol/L, *m* = 0.02–0.1 g; for nitrobenzene—*c_o_* = 0.5–6.18 mmol/L, *m* = 0.02–0.08 g; and for 4-nitrobenzoic acid—*c_o_* = 0.5–1 mmol/L, *m* = 0.01–0.1 g. After establishing equilibrium in the activated carbon–adsorbate system, absorption measurements in the UV range were performed using a Varian Cary 100 UV–vis spectrophotometer (Varian, Melbourne, Victoria, Australia) to determine the solute equilibrium concentration. The amount of adsorbed substance was determined from the mass balance equation:

(1) $$a_{eq}=\frac{\left(c_{o}-c_{eq}\right)\cdot V}{m}$$

where *a_eq_* is the adsorption of an adsorbate at equilibrium, *c_eq_* is the equilibrium concentration, *V* is the solution volume, and *m* is the adsorbent mass.

The obtained experimental adsorption data were analyzed by using the generalized Langmuir (GL) equation [31], which describes the systems showing energetic heterogeneity:

(2) $$\theta ={\left[\frac{{\left(Kc_{eq}\right)}^{n}}{1+{\left(Kc_{eq}\right)}^{n}}\right]}^{m/n}$$

In the above, *θ* = *a_eq_*/*a_m_* is the solute relative adsorption, *a_m_* is an adsorption capacity, *m* and *n* are the heterogeneity parameters characterizing the shape (width and asymmetry) of the adsorption energy distribution functions (0 ≤ *m*, *n* ≤ 1), and *K* is the equilibrium constant determining the position of the distribution function on the energy axis.

The GL equation [31] may be reduced to known adsorption isotherms for specific values of the heterogeneity parameters m and n. For *m* = *n* ϵ (0,1), it takes the form of the Langmuir–Freundlich (LF) isotherm; for *n* = 1 and *m* ϵ (0,1), it becomes the generalized Freundlich (GF) isotherm; for *m* = 1 and *n* ϵ (0,1), it is the Tóth (T) isotherm; and for *m* = *n* = 1, it is reduced to the Langmuir (L) isotherm.

## 4. Conclusions

Four samples of active carbons of differentiated surface properties were obtained in the processes of chemical modification. The surface properties of the obtained adsorbents are differentiated with regard to the amount and type of surface functional groups. The influence of acid/base properties of active carbons on the adsorption of various organic solutes from dilute aqueous solutions was investigated. The weakest adsorption was observed on the F-NA carbon, oxidized with nitric acid. The relatively high adsorption found for the deashed F-NM sample was a result of increasing the overall pore volume and adsorption capacity of this material by removing ash, partly blocking micropores. The commercial F-400 carbon, containing some ash, and the F-SA carbon, modified with sulfuric acid, exhibited slightly reduced sorption properties. The F-Oz carbon modified by ozone revealed relatively higher adsorption efficiency in comparison with other oxidized carbons. Thus, the oxidation process leads to a reduction in the adsorption of organic substances; the type of modification used significantly affects the adsorption efficiency. For nitrobenzene, adsorption capacity was the highest for deashed carbon F-NM (5.11 mmol/g), it decreased strongly for the material oxidized with nitric acid (3.59 mmol/g), and it decreased only slightly for F-Oz modified with ozone (4.85 mmol/g).

In the case of toluene, nitrobenzene, and 4-nitrobenzoic acid, similar adsorption behavior was observed for four carbons (F-400, F-NM, F-NA, and F-SA), with significantly higher adsorption of toluene compared to the other compounds (T > NT > NB > 4-NBA), whereas for the carbon F-Oz oxidized with ozone, the highest adsorption was found for nitrotoluene. The differences in adsorption uptakes were found to be a result of the differentiation in adsorbate hydrophobicity determined by their solubilities, the molecular size, and the effect of adsorbate functional groups on the interactions with a carbon surface. The varied influence of the number and nature of surface oxygen groups and their variability over time in applications in liquid systems containing different compounds must be taken into account when designing application solutions.

## Figures and Tables

**Table 1:** Selected physicochemical properties of the carbons investigated.

Carbon Sample *	S_BET_, m^2^/g	V_mi_, cm^3^/g	V_t_, cm^3^/g	Total Oxygen wt.%	pH of Carbon Slurry	HCl Uptake meq/g	NaOH Uptake meq/g	Δm/m, %	
								150–400 °C	400–600 °C
F-NM	1050	0.512	0.628	2.26	7.40	0.020	0.220	0.82	0.33
F-NA	975	0.457	0.554	17.54	3.51	0.291	2.212	5.77	4.25
F-SA	940	0.442	0.542	3.78	3.75	0.178	0.813	3.13	0.45
F-Oz	935	0.441	0.540	17.34	4.53	0.203	0.808	2.77	1.77

* F—Filtrasorb-400, NM—non-modified, NA—nitric acid, SA—sulfuric acid, Oz—ozone.

**Table 2:** Surface chemical composition of F-400 activated carbon modified samples determined by SEM-EDS.

Element	F-NM	F-NA	F-SA	F-Oz
	%wt.			
C	95.26	82.07	90.98	81.94
O	2.26	17.54	3.78	17.34
S	1.47	0.27	3.92	0.42
Cl	1.02	0.11	1.32	0.31

**Table 3:** Physicochemical characteristics of adsorbates.

Adsorbate	Molecular Weight [g/mol]	Solubility in H_2_O [mmol/L]	Ionization Constant (pKa)
toluene (T)	92.14	5.43	-
4-nitrotoluene (NT)	137.14	3.21	-
nitrobenzene (NB)	123.11	15.43	-
4-nitrobenzoic acid (4-NBA)	167.12	2.51	3.44

**Table 4:** Parameters of the generalized Langmuir equation characterizing the adsorption of toluene (T), nitrotoluene (NT), nitrobenzene (NB), and nitrobenzoic acid (4-NBA) from dilute aqueous solutions on commercial F-400 and the modified carbons.

Adsorption System	Isotherm Type	*a_m_*	*m*	*n*	*K*	*R* ^2^
T/F-400	L	5.28	1	1	8.22	0.88
T/F-NM	GF	6.20	0.21	1	0.42	0.96
T/F-NA	GF	4.20	0.23	1	0.54	0.83
T/F-SA	GF	5.10	0.39	1	4.13	0.94
T/F-Oz	GF	5.51	0.32	1	0.72	0.94
NT/F-400	GF	3.45	0.33	1	5.14	0.98
NT/F-NM	GF	3.75	0.87	1	15.9	0.94
NT/F-NA	GF	3.90	0.41	1	0.54	0.95
NT/F-SA	GF	3.93	0.33	1	2.05	0.93
NT/F-Oz	GF	5.02	0.32	1	5.10	0.82
NB/F-400	GL	4.01	0.66	0.72	6.82	0.99
NB/F-NM	GL	5.11	0.36	0.53	1.56	0.99
NB/F-NA	GL	3.59	0.39	0.65	0.12	0.99
NB/F-SA	GF	4.12	0.34	1	1.38	0.99
NB/F-Oz	GL	4.85	0.47	0.45	3.75	0.99
4-NBA/F-400	GF	3.02	0.30	1	1.15	0.81
4-NBA/F-NM	GF	3.50	0.28	1	1.20	0.93
4-NBA/F-NA	GF	2.84	0.30	1	0.33	0.98
4-NBA/F-SA	GL	2.94	0.58	0.61	32.03	0.95
4-NBA/F-Oz	GL	3.82	0.44	0.40	6.32	0.94

**Table 5:** Comparison of the results of adsorption studies for selected aromatic compounds on modified activated carbons.

Activated Carbon	S_BET_ [m^2^/g]	Adsorption Capacity [mmol/g]				Ref.
		**Adsorbate**				
		Phenol	Chlorophenol	Nitrophenol	Aniline	
Norit RS 08						[30]
-deashed, heat treated (He 900 °C)	1080	3.29	3.19	2.52	3.20	
and oxidized with HNO_3_ (10 wt%)	873	1.75	1.85	1.78	2.01	
		Phenol	Nitrobenzene	Aniline		
Norit C Gran	1317	1.30	1.70	1.70		[29]
Norit ROX 08	1047	2.10	1.97	1.96		
-oxidized with HNO_3_ (5 M)	883	1.90	1.60	2.10		
-and heat treated (H_2_ 700 °C)	940	1.82	1.53	1.79		
		Toluene	Nitrotoluene	Nitrobenzene	4-Nitrobenzoic acid	
F-400	997	5.28	3.45	4.01	3.02	This work
-deashed	1050	6.20	3.75	5.11	3.50	
-oxidized with conc. HNO_3_	975	4.20	3.90	3.59	2.84	
-oxidized with conc. H_2_SO_4_	940	5.10	3.93	4.12	2.94	
-oxidized with O_3_	935	5.51	5.02	4.85	3.82	

## Data Availability

The data are available by the corresponding author.

## Supplementary Materials

The following supporting information can be downloaded at: https://www.mdpi.com/article/10.3390/molecules30183810/s1, Figure S1: Comparison of adsorption isotherms of studied adsorbates from aqueous solutions on. carbons: F-400, F-NM, F-NA, F-SA, F-Oz (influence of adsorbate properties).
