# Supplementary material for: The Effect of Oxidative Modification of Activated Carbon on Adsorption of Aromatic Compounds from Aqueous Solutions
Source: Molecules. 2025 Sep 19;30(18):3810. doi: 10.3390/molecules30183810 (PMC12472693; doi:10.3390/molecules30183810)
Supplement: Supplementary file 1 [file molecules-30-03810-s001.zip › molecules-3849958-supplementary.pdf]

Supplementary material for the paper

Title: The effect of oxidative modification of activated carbon on adsorption of aromatic compounds from aqueous solutions

Authors: Anna Derylo-Marczewska [1]*, Andrzej Swiatkowski [2], Grzegorz Trykowski [3] and Stanislaw Biniak [3]

Affiliation:

[1] Institute of Chemical Sciences, Faculty of Chemistry, Maria Curie-Sklodowska University, M. Curie-Sklodowska Sq. 3, 20-031 Lublin, Poland

[2] Institute of Chemistry, Military University of Technology, Kaliskiego 2, Warsaw 00-908, Poland

[3] Faculty of Chemistry, N. Copernicus University, 87-100 Torun, Poland

* Correspondence: anna.derylo-marczewska@mail.umcs.pl (A.D.-M.) Tel.: +48-8153-755-49

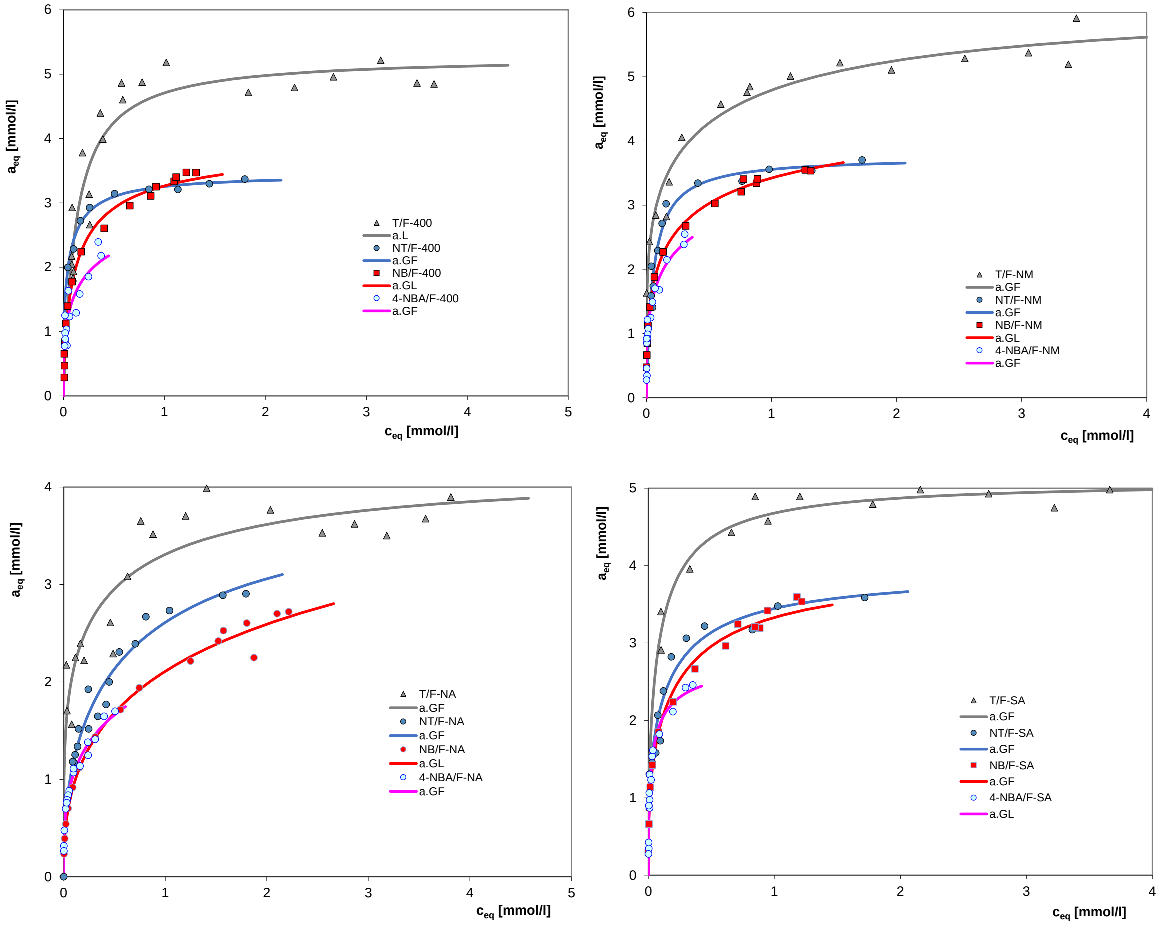

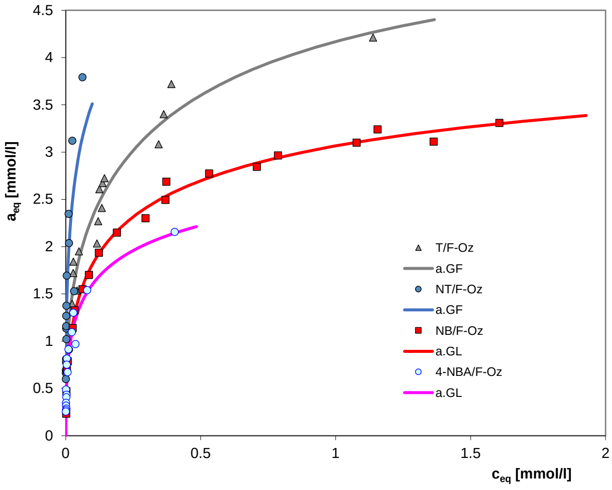

Figure S1. Comparison of adsorption isotherms of studied adsorbates from aqueous solutions on carbons: F-400, F-NM, F-NA, F-SA, F-Oz (influence of adsorbate properties).
